# Association of Plasma Claudin-5 with Age and Alzheimer Disease

**DOI:** 10.3390/ijms25031419

**Published:** 2024-01-24

**Authors:** Keisuke Tachibana, Ryuichi Hirayama, Naoyuki Sato, Kotaro Hattori, Takashi Kato, Hiroyuki Takeda, Masuo Kondoh

**Affiliations:** 1 Graduate School of Pharmaceutical Sciences, Osaka University, Suita 565-0871, Osaka, Japan; nya@phs.osaka-u.ac.jp; 2 Graduate School of Medicine, Osaka University, Suita 565-0871, Osaka, Japan; rhirayama@nsurg.med.osaka-u.ac.jp (R.H.); nsato@ncgg.go.jp (N.S.); 3 Department of Aging Neurobiology, Center for Development of Advanced Medicine for Dementia, National Center for Geriatrics and Gerontology, Obu 474-8511, Aichi, Japan; 4 Department of Bioresources, Medical Genome Center, National Center of Neurology and Psychiatry, Kodaira 187-8551, Tokyo, Japan; hattori@ncnp.go.jp; 5 Department of Clinical and Experimental Neuroimaging, National Center for Geriatrics and Gerontology, Obu 474-8511, Aichi, Japan; tkato@ncgg.go.jp; 6 Proteo-Science Center, Ehime University, Matsuyama 790-8577, Ehime, Japan; takeda.hiroyuki.mk@ehime-u.ac.jp

**Keywords:** blood–brain barrier, claudin-5, tight junction, biomarker, dementia, Alzheimer disease

## Abstract

The blood–brain barrier (BBB) plays pivotal roles in synaptic and neuronal functioning by sealing the space between adjacent microvascular endothelial cells. BBB breakdown is present in patients with mild cognitive impairment (MCI) or Alzheimer disease (AD). Claudin-5 (CLDN-5) is a tetra-spanning protein essential for sealing the intercellular space between adjacent endothelial cells in the BBB. In this study, we developed a blood-based assay for CLDN-5 and investigated its diagnostic utility using 100 cognitively normal (control) subjects, 100 patients with MCI, and 100 patients with AD. Plasma CLDN-5 levels were increased in patients with AD (3.08 ng/mL) compared with controls (2.77 ng/mL). Plasma levels of phosphorylated tau (pTau181), a biomarker of pathological tau, were elevated in patients with MCI or AD (2.86 and 4.20 pg/mL, respectively) compared with control subjects (1.81 pg/mL). In patients with MCI or AD, plasma levels of CLDN-5—but not pTau181—decreased with age, suggesting some age-dependent BBB changes in MCI and AD. These findings suggest that plasma CLDN-5 may a potential biochemical marker for the diagnosis of AD.

## 1. Introduction

Currently, more than 50 million people worldwide have some form of dementia, and this population is anticipated to grow to 78 million by 2030 [1]. Alzheimer disease (AD) is the most common cause of dementia. AD is a progressive disease, the preclinical stage of which includes mild cognitive impairment (MCI). Current therapeutic strategies for AD aim to reduce cognitive decline and involve various cognitive enhancers (e.g., donepezil, memantine) and disease modifiers (e.g., aducanumab, lecanemab) [2,3]. To maximize therapeutic efficacy, identifying people at risk of AD and initiating treatment during preclinical or early AD is crucial.

The clinical symptoms of AD reflect the associated progressive brain atrophy and subsequent cognitive decline. However, various molecular pathologies in the brain—including the accumulation of amyloid protein, phosphorylation of tau protein, and breakdown of the blood–brain barrier (BBB)—typically precede clinical symptoms [4,5]. The diagnosis of AD rests on measuring the accumulation of amyloid or tau proteins in the brain or their spillover into the cerebrospinal fluid (CSF) or blood. However, the collection of CSF typically involves lumbar puncture, which is an invasive procedure. An alternative method is positron emission tomography (PET) using amyloid and tau probes, which detects deposits of these proteins in the brain. Although PET imaging is minimally invasive, it is not widely used because of its expense [6]. Blood-based tests have recently gained attention as alternative diagnostic options because they are minimally invasive and cost-effective. Blood tests for amyloid and phosphorylated tau proteins have been developed and are used in people who have symptoms of MCI or AD [7,8].

The BBB functions as a selective gate for the uptake of essential molecules from blood into the brain and the excretion of harmful molecules from the brain into blood via transporters and receptors on cellular membranes [9]. In addition, the BBB prevents the influx of blood-borne neurotoxins, cells, and pathogens into the brain because of the formation of tight junctions (TJs) in the intercellular space between adjacent macrovascular endothelial cells. Loss of BBB integrity has been observed in neuroinflammatory disorders [5], and patients with early AD demonstrate BBB leakage [10]. In addition, patients with early cognitive dysfunction show BBB breakdown in the hippocampus, which occurs independently of brain accumulation of amyloid and tau [11]. These several findings indicate that BBB TJ-sealing components might be impaired in MCI- and AD-related pathology.

Claudin is a tetra-transmembrane protein and an essential component of the TJ seal [12]. In mammals, claudins comprise a protein family of 27 members, and claudin-5 (CLDN-5) is a pivotal TJ-sealing protein in the BBB [12,13]. Blood CLDN-5 levels are elevated in patients with multiple sclerosis [14], and loss of CLDN-5 was accompanied by BBB breakdown in a mouse model of autoimmune encephalomyelitis [15]. These findings suggested to us that blood CLDN-5 levels might be altered in patients with MCI and AD compared with their cognitively normal individuals. To investigate this idea, we first used a monoclonal antibody (mAb) that recognizes the extracellular loops of human CLDN-5 to develop a highly sensitive single-molecular array (Simoa) assay, which is more sensitive than conventional immunoassays. We then used this assay to qualitatively investigate plasma CLDN-5 levels in cognitively normal adults and patients with MCI or AD and considered the clinical implications of plasma CLDN-5 levels in AD from the point of view of correlations of plasma CLDN-5 levels with age and a phosphorylated tau protein (pTau-181), a biomarker of cognitive decline [7].

## 2. Results

### 2.1. Establishment of an Immunoassay System Using Simoa

To investigate whether plasma CLDN-5 protein levels change in patients with MCI and AD, we established a sensitive immunoassay system using Simoa technology and an anti-CLDN-5 mAb against the second extracellular loop domain of human CLDN-5 [16].

**Figure 1:**
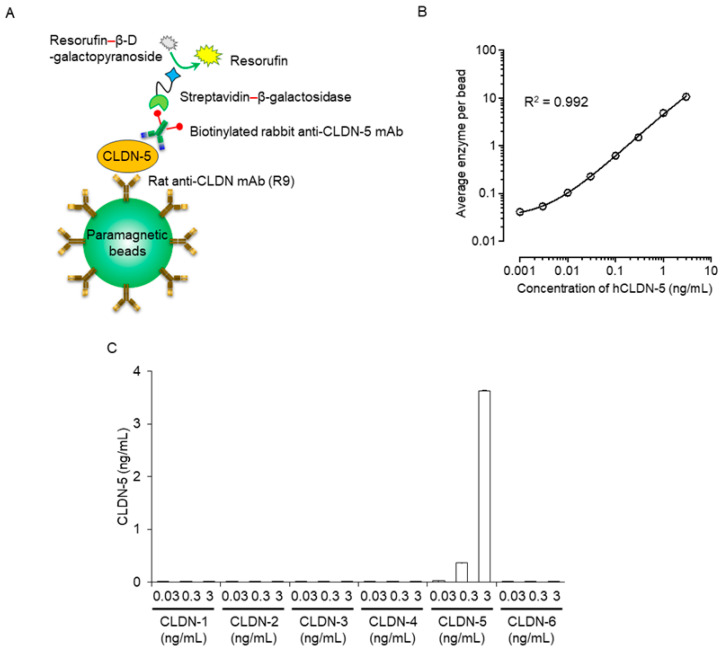
Sensitivity and specificity of a plasma human claudin (CLDN) 5 protein detection system using Simoa. (**A**) Schematic illustration of singleplex detection of human CLDN-5 using Simoa. First, the capture CLDN-5 monoclonal antibody (mAb) (clone R9) was immobilized on carboxylated paramagnetic beads. The CLDN-5 protein was captured on the antibody-coupled beads and then sandwiched with the biotinylated detector CLDN-5 mAb. Next, the beads were labeled with streptavidin–β-galactosidase. Finally, the immunocomplex beads were mixed with the enzyme substrate resorufin β-D-galactopyranoside and loaded onto the disc microarray. Fluorescent signals were analyzed automatically to obtain the average enzyme amount per bead. (**B**) Response curve of the Simoa assay for the detection of human CLDN-5. Data are given as mean ± standard deviation (n = 5). Standard curves were generated using a four-parameter logistic model, and R-squared values were calculated. (**C**) Analysis of the specificity of CLDN-5 detection using Simoa. Proteoliposomes containing human CLDN-1 to -6 were tested at concentrations of 0.03, 0.3, and 3 ng/mL. Data are given as mean ± standard deviation (n = 3).

To this end, we chemically coated magnetic beads with antibodies recognizing the extracellular loop domain of CLDN-5. Samples were incubated with anti-CLDN-5-coated beads; the bound CLDN-5 was sandwiched with a biotinylated anti-CLDN-5 antibody against the intracellular domain of CLDN-5; and the complexes were incubated with streptavidin β-galactosidase, followed by the addition of chemiluminescent substrate (Figure 1A). The generated chemiluminescence signal was measured through digital scanning. The Simoa immunoassay for CLDN-5 had a dynamic range of 0.01 to 3 ng/mL, with a limit of detection of 0.001 ng/mL; a regression line with good fit (R^2^ = 0.992); a low coefficient of variability; high accuracy; and high CLDN-5 specificity (Figure 1B,C, Table 1, Supplementary Table S1).

### 2.2. Association of Plasma CLDN-5 Levels with the Stages of Cognitive Decline

**Figure 2:**
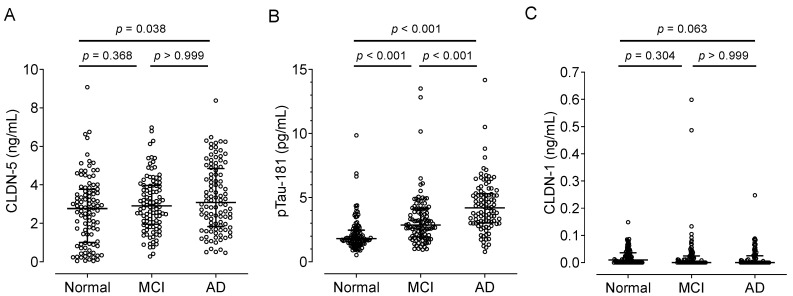
Comparison of plasma CLDN-5, pTau-181 or CLDN-1 levels among cognitively normal controls and patients with mild cognitive impairment (MCI) or Alzheimer disease (AD). Plasma samples were collected from cognitively normal individuals, MCI patients, and AD patients in the NCGG Biobank (n = 100 per group). Plasma (**A**) CLDN-5, (**B**) pTau-181, and (**C**) CLDN-1 levels were measured by using a Simoa system as described in the Materials and Methods section. The horizontal bar indicates the median value, and the error bars the 25th and 75th percentiles of each group. The statistical analysis was performed using the Kruskal–Wallis nonparametric ANOVA test along with the Dunn multiple-comparisons test.

We obtained plasma samples from cognitively healthy (control) adults and patients with MCI or AD from Japan’s National Center for Geriatrics and Gerontology Biobank (Table 2). The median plasma CLDN-5 level increased with cognitive impairment: 2.77, 2.91, and 3.08 ng/mL in the control, MCI, and AD groups, respectively (Figure 2A). Plasma CLDN-5 levels were significantly higher in AD patients than in cognitively healthy adults (*p* < 0.05). As previously reported, plasma pTau-181 levels, a biomarker of tau phosphorylation, showed a positive relationship with the stages of cognitive decline (Figure 2B) [7]. We also established the measurement of the plasma concentrations of CLDN-1 (Supplementary Figure S1, Supplementary Tables S2 and S3), one of the TJ proteins of the BBB with minor contributions [17]. There were no differences in plasma CLDN-1 concentrations among these groups: the medians of plasma CLDN-1 concentrations were 0.01, 0.00, and 0.00 ng/mL in the control, MCI, and AD groups, respectively (Figure 2C).

**Figure 3:**
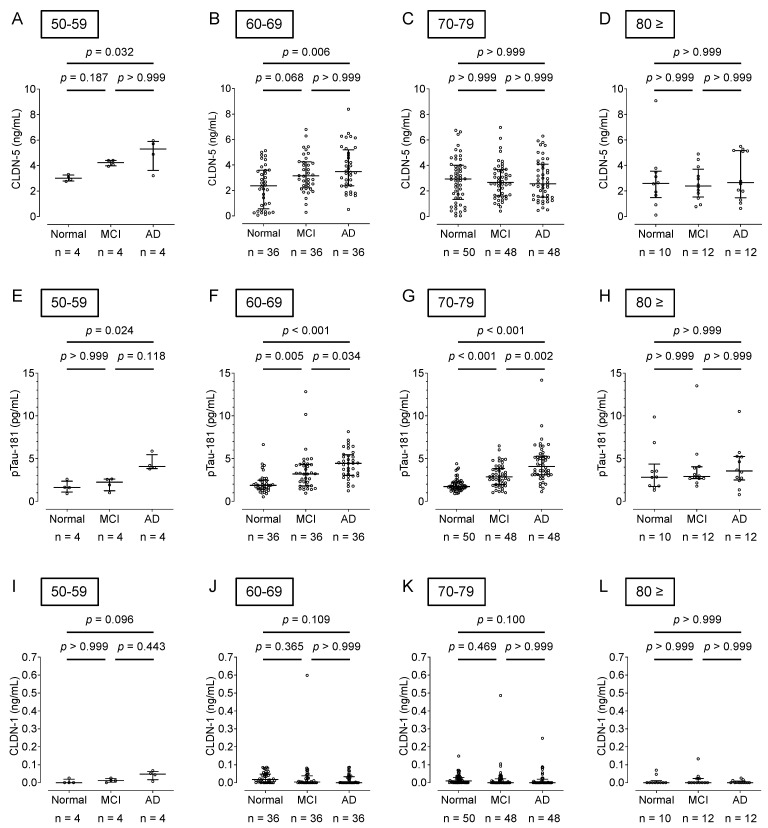
Comparison of the plasma CLDN-5, pTau-181, and CLDN-1 levels by age groups in cognitively normal controls and patients with MCI or AD. Plasma CLDN-5 (**A**–**D**), pTau-181 (**E**–**H**), and CLDN-1 (**I**–**L**) levels from cognitively normal controls and patients with MCI or AD, shown in Figure 2, were replotted according to age groups. The horizontal bar indicates the median value and the error bars the 25th and 75th percentiles of each group. The statistical analysis was performed using the Kruskal–Wallis nonparametric ANOVA test along with the Dunn multiple-comparisons test.

A comparison of plasma CLDN-5 levels by age showed that AD patients in their 50s and 60s had significantly higher levels than cognitively healthy adults (*p* < 0.05) (Figure 3A–D). Plasma pTau-181 levels were significantly higher in AD patients aged 50 to 70 years than in cognitively healthy adults (*p* < 0.05) (Figure 3E–H). There was no difference in plasma CLDN-1 levels between AD patients and cognitively healthy adults in any age group over 50 years (Figure 3I–L).

**Figure 4:**
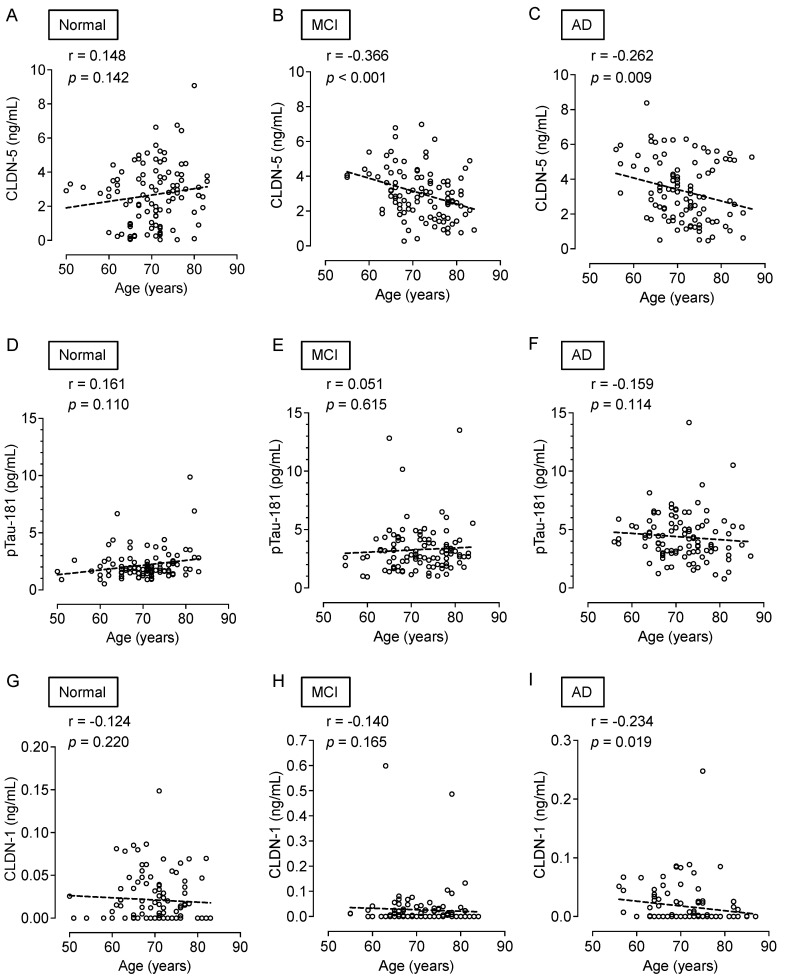
Association of the plasma CLDN-5, pTau-181, and CLDN-1 levels with age. Spearman rank-correlation analyses of plasma CLDN-5 concentration and age are shown for (**A**) cognitively normal controls, (**B**) patients with MCI, and (**C**) patients with AD. Those of plasma pTau-181 and age are shown for (**D**) cognitively normal controls, (**E**) patients with MCI, and (**F**) patients with AD, and those of plasma CLDN-1 concentration and age are shown in (**G**) cognitively normal controls, (**H**) patients with MCI, and (**I**) patients with AD.

While cognitively normal subjects did not show an age-dependent change in CLDN-5 (Figure 4A), plasma CLDN-5 levels were higher in younger than in older patients in both the MCI (*p* < 0.001) and AD (*p* < 0.01) groups (Figure 4B,C). However, neither MCI nor AD patients showed age-related changes in plasma pTau-181 levels (Figure 4D–F). Plasma CLDN-1 levels tended to decrease with age in all cognitive function groups (Figure 4G–I).

**Figure 5:**
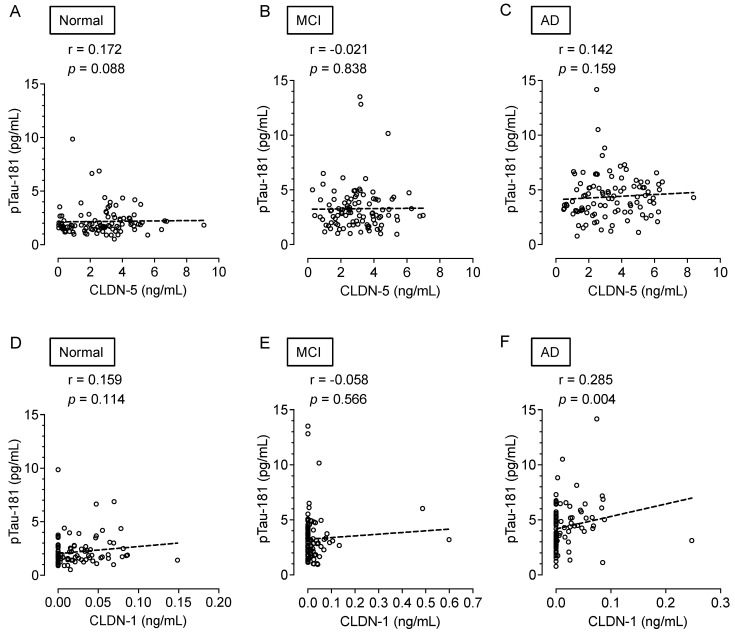
Association of the plasma CLDN-5 or CLDN-1 levels with plasma pTau-181 level. Spearman rank-correlation analyses of plasma CLDN-5 and pTau-181 concentrations are shown for (**A**) cognitively normal controls, (**B**) patients with MCI, and (**C**) patients with AD. Those of plasma CLDN-1 and pTau-181concentrations are shown for (**D**) cognitively normal controls, (**E**) patients with MCI, and (**F**) patients with AD.

Finally, we investigated the correlation between plasma CLDN levels and plasma pTau-181 levels, a biomarker for the diagnosis of AD. CLDN-5 levels were not associated with pTau-181 levels in any of the groups (Figure 5A–C). Plasma CLDN-1 levels in AD patients correlated with pTau-181 levels (*p* = 0.004) (Figure 5D–F). These data indicate that CLDN-5 and pTau-181 are independent biomarkers in the plasma.

## 3. Discussion

A body of evidence supports the notion that microvascular degeneration is associated with the pathophysiology of AD [5,18]. In the current study, we investigated associations of the plasma concentration of CLDN-5, a pivotal TJ-sealing protein in the microvascular endothelial cells of the BBB, with MCI and AD. We found that plasma CLDN-5 levels were higher in patients with AD than in cognitively healthy adults, and plasma CLDN-5 levels were higher in younger than in older patients in both the MCI and AD groups.

Breakdown of the BBB, which is associated with CNS diseases, is accompanied by the invasion of leukocytes and activation of astrocytes [5]. The matrix metalloproteinases (MMPs) secreted by these invading leukocytes have been shown to lead to the degradation of CLDN-5 in the BBB of mice [19]. In a rat ischemic model, MMPs secreted from astrocytes likewise degraded CLDN-5 in the BBB [20]. In addition, the number of pericytes in the BBB was greater in patients with AD compared with cognitively healthy peers, perhaps reflecting a response to endothelial breakdown [21]. As is similar to our findings for patients with MCI or AD, circulating CLDN-5 levels are elevated in other CNS diseases, including ischemic stroke, bipolar disorder, and obsessive–compulsive disorder [22,23,24]. The CLDN-5 circulating in blood might be derived from the endothelial cells in the BBB.

Interestingly, we found a significant negative association of plasma CLDN-5 level with age in MCI and AD. Ultrastructural analysis of TJ seals in the BBB did not reveal normal age-associated changes [25]. In contrast, magnetic resonance imaging showed that people with no cognitive impairment had an age-dependent progressive loss of BBB integrity in the hippocampus, which plays a pivotal role in learning and memory and which is damaged in early AD [26]. In another study, adults with early cognitive dysfunction developed brain capillary damage and BBB breakdown in the hippocampus [11]. Furthermore, the number of pericytes in the BBB was increased in patients with AD compared with age-matched controls without dementia [21]. In the current study, CLDN-5 levels were higher in younger than in older patients in both the MCI and AD groups. This is consistent with an analysis of autopsied brains which reported that the CLDN-5 level decreases with AD progression [27]. Because reactive astrocytes and endothelial cells in the BBB in AD produce MMPs [28], prolonged activation of MMPs might lead to the degradation of CLDN-5 and, thus, lower plasma CLDN-5 levels in older compared with younger patients with cognitive deficits.

We acknowledge several limitations of this study. First, the source of the circulating CLDN-5 remains unknown. CLDN-5 is expressed in endothelial cells throughout the body [29], and at greater levels by CNS endothelial cells than by those outside of the CNS [30]. Endothelial-cell-specific CLDN-5^+/−^ mice, whose CLDN-5 protein levels were 50% lower than those of CLDN-5^+/+^ mice, had learning and memory deficits [31,32]. Downregulation of CLDN-5 in the prefrontal cortex promoted anxiety-like and depression-like behaviors in mice [33]. Suppression of CLDN-5 in the hippocampus impaired spatial recognition memory in mice [32]. Together, these data suggest that local suppression of CLDN-5 levels may affect the onset of AD. Second, we did not investigate comorbidities of AD. Patients with chronic kidney diseases have higher risks of dementia and MCI than members of the general population [34]. In addition, heart failure is a risk factor for AD [35,36], and epidemiologic studies indicate that AD is seen more frequently in people with asthma [37]. The apolipoprotein E ɛ4 allele is associated with a high risk of AD [38]. Smoking and hypertension are also risk factors for AD [39,40]. Moreover, high serum iron and aluminum levels may provide a background for promoting the onset of AD [41]. The influences of these complications need to be defined to clarify the clinical significance of increased circulating CLDN-5 levels. Third, we did not address whether the detected CLDN-5 protein is the post-translationally modified form. CLDN-5 has multiple glycosylation, phosphorylation, and ubiquitination sites, which may be associated with enhancing the permeability of the BBB [42,43]. Fourth, multiple sclerosis (MS) is a neurodegenerative disease showing increased CLDN-5 levels [44], whereas major depressive disorder and schizophrenia are psychiatric disorders associated with increased and decreased plasma and serum CLDN-5 levels, respectively [45,46]. Cognitive impairment occurs in approximately half of patients with MS [47]. Although patients with MS or these psychiatric disorders were excluded from this study, plasma CLDN-5 levels should be investigated in patients with AD and other central nervous system-related diseases in order to understand the clinical utility of plasma CLDN-5 levels. Further investigation into the clinical meaning and utility of circulating CLDN-5 will be useful for the diagnosis of MCI and AD. In conclusion, CLDN-5 may be a potential biochemical marker of MCI and AD.

## 4. Materials and Methods

### 4.1. Clinical Samples

Samples of human plasma were obtained from the national biobank of the National Center for Geriatrics and Gerontology (Aichi, Japan) [48]. To explore the diagnostic utility of a plasma CLDN-5 assay, we obtained plasma samples from cognitively normal subjects and those with MCI or AD from the National Center for Geriatrics and Gerontology Biobank (Table 2). Basic demographic data, including age, gender, diagnosis, and Mini-Mental State Examination score, were obtained from the biobank. To avoid analytical bias, the analysis of plasma CLDN-5 was carried out in a blinded manner, and the sample identities were only revealed from the biobank after the analysis. This study was approved by the ethics committees of Osaka University (protocol no. Yakuhito2020-1-4) and the National Center for Geriatrics and Gerontology (20TB6). All methods were utilized in accordance with the relevant guidelines and regulations.

### 4.2. Purification of Anti-CLDNs mAbs

Purified rat anti-CLDN-5 mAbs (clone R9) and mouse anti-CLDN-1 mAbs (clone 2C1) were prepared as described previously [49,50]. Briefly, hybridoma cells were cultured in Hybridoma SFM medium (Thermo Fisher Scientific, Waltham, MA, USA) containing 10% BM Condimed H1 (Roche, Mannheim, Germany). The anti-CLDN mAbs were purified from the culture media using Protein G Sepharose 4 Fast Flow columns (Cytiva, Marlborough, MA, USA). Buffer exchange was performed using phosphate-buffered saline (PBS, pH 7.4) and centrifugal filter tubes with a molecular weight cutoff of 100 kDa (Amicon Ultra-100K, Merck Millipore, Burlington, MA, USA). Purified mAb was sterilized by filtering it through a 0.22 µm filter, and then stored at −30 °C. The concentration of mAb was quantified using a BCA Protein Assay kit (Nacalai Tesque, Kyoto, Japan) with bovine serum albumin as the standard.

### 4.3. Preparation of CLDNs

Cell-free synthesis of CLDN recombinant proteins was performed with the bilayer-dialysis method as described previously, with minor modifications [51,52]. Briefly, in vitro transcription was performed using SP6 RNA polymerase (CellFree Sciences, Matsuyama, Japan). The translation reaction mixture (500 µL) containing mRNA (25%), WEPRO 7240 wheat germ extract (25%; CellFree Sciences), creatine kinase (40 μg/mL; Roche), and asolectin liposomes (10 mg lipids/mL) was overlaid with 2 mL of SUB-AMIX SGC dialysis solution (CellFree Sciences) in a 10-K MWCO Slide-A-Lyzer MINI dialysis device (Thermo Fisher Scientific); the cup was then immersed in 3.5 mL of SUB-AMIX SGC solution and incubated at 16 °C for 24 h. Cell-free synthesized proteoliposomes were collected by centrifugation at 20,000× *g* for 10 min at 4 °C, and the resultant pellet was suspended in HBS buffer (20 mM Hepes-NaOH, pH 7.2, 150 mM NaCl). Proteoliposomes were washed three times with HBS, then finally resuspended in 125 μL of HBS buffer. To solubilize the proteoliposomes, 125 μL of a solution (20 mM Hepes-NaOH, pH 7.2, 150 mM NaCl, 1% n-dodecyl-β-D-maltoside (Dojindo, Kumamoto, Japan), 10% glycerol (Nacalai Tesque), 1 mM DTT) was mixed with the 125 µL proteoliposomes suspension. The mixture was rotated gently at 4 °C for 1 h, sonicated for 3 min at 4 °C using an ultrasonic disruptor (SONIFIER model 450 Advanced, Branson, CT, USA), and then centrifuged at 17,800× *g* and 4 °C for 15 min. The supernatant was aliquoted in small portions, frozen in liquid nitrogen, and stored at –80 °C.

### 4.4. Simoa Assay for CLDNs

Plasma CLDN-5 and CLDN-1 concentrations were determined with Simoa technology and a Homebrew assay kit (Quanterix, Billerica, MA, USA) in accordance with the manufacturer’s instructions. In the first step of the assay, carboxylated paramagnetic beads were activated using 1-ethyl-3-(3-dimethylaminopropyl) carbodiimide hydrochloride (Thermo Fisher Scientific), followed by incubation with rat anti-CLDN-5 mAb (clone R9) or mouse anti-CLDN-1 mAb (clone 2C1) for 2 h at 4 °C with rotation. After blocking of the antibody-coupled beads with Bead Blocking Buffer (Quanterix), the beads were resuspended with Bead Diluent (Quanterix) and stored at 4 °C until further use. For biotinylation of the detection antibodies, bovine serum albumin and azide-free rabbit anti-CLDN-5 mAb (Abcam, Cambridge, UK) or rabbit anti-CLDN-1 mAb (Abcam) were mixed with NHS-PEG4-Biotin (Thermo Fisher Scientific) and reacted at room temperature for 30 min. The biotinylated antibody was buffer-exchanged into PBS using an Amicon centrifugal filter with a cutoff value of 50 kDa (Merck Millipore), then stored at –20ºC until use.

Singleplex Simoa assays were performed by using the HD-X Analyzer (Quanterix, Billerica, MA, USA). Human plasma samples and standard human CLDN-5 or human CLDN-1 proteins were incubated with the antibody-coated capture beads (250,000 beads per test for CLDN-5 or 187,500 beads per test for CLDN-1) for 15 min. After washing, the beads were incubated with biotinylated detector antibody for 5.25 min. After a series of washes, streptavidin–β-galactosidase (Quanterix) solution was added and incubated for 5.25 min. The beads were washed again, mixed with the enzyme substrate resorufin β-D-galactopyranoside (Quanterix), and loaded onto the disc microarray. The array was sealed with oil, and the average enzyme per bead values were calculated using the software in the HD-X Analyzer (version 3.0) [53].

Plasma pTau-181 concentrations were determined using Simoa technology and the pTau-181 Advantage V2 kit (Quanterix) on the HD-X Analyzer in accordance with the manufacturer’s instructions.

### 4.5. Data Analysis

Data are expressed as mean ± standard deviation. Standard curves for determining unknown sample concentrations were fitted using the Prism (version 9, GraphPad Software, Boston, MA, USA) four-parameter logistic fit. Statistical analyses were performed using the Dunn multiple-comparison test (Prism, GraphPad Software), and correlations were calculated using Spearman rank correlation analysis (Prism, GraphPad Software).

## Figures and Tables

**Table 1:** Precision and accuracy of quantification of human CLDN-5 concentration series.

Theoretical Concentration (ng/mL)	Actual Concentration (ng/mL)					Mean (ng/mL)	SD	CV (%)	Accuracy
	1	2	3	4	5				
0	ND	ND	ND	0.000	ND	0.000	NA	NA	NA
0.001	0.001	0.001	0.001	0.001	0.001	0.001	0.000	13.80	117.18
0.003	0.003	0.004	0.003	0.003	0.003	0.003	0.000	15.51	95.37
0.01	0.010	0.011	0.011	0.010	0.009	0.010	0.001	7.61	102.21
0.03	0.029	0.033	0.030	0.029	0.029	0.030	0.002	5.74	100.19
0.1	0.103	0.108	0.096	0.105	0.090	0.100	0.007	7.47	100.27
0.3	0.290	0.315	0.293	0.280	0.208	0.277	0.041	14.73	92.46
1	1.094	1.152	1.082	1.083	1.048	1.092	0.038	3.47	109.19
3	3.008	3.180	2.888	2.517	2.981	2.915	0.246	8.44	97.16

CV, coefficient of variation; NA, not applicable; ND, not detected; SD, standard deviation.

**Table 2:** Characteristics of patients who provided the plasma samples from Japan’s NCGG Biobank used in this study.

Characteristic	Cognitively Normal (n = 100)	MCI (n = 100)	AD (n = 100)
Age, y	70.3 ± 6.7	71.7 ± 6.6	71.5 ± 6.7
Female, n (%)	50 (50.0)	50 (50.0)	50 (50.0)
MMSE score	29.5 ± 0.7	22.2 ± 1.9	16.0 ± 3.8

AD, Alzheimer disease; MCI, mild cognitive impairment; MMSE, Mini-Mental State Examination. Data are shown as mean ± S.D.

## Data Availability

Data are contained within the article and supplementary materials.

## Supplementary Materials

The following supporting information can be downloaded at: https://www.mdpi.com/article/10.3390/ijms25031419/s1.

## References

[1] Gauthier S., Webster C., Servaes S., Morais J., Rosa-Neto P. (2022). World-Alzheimer-Report 2022: Life after Diagnosis: Navigating Treatment, Care and Support.

[2] Hameed S., Fuh J.L., Senanarong V., Ebenezer E.G.M., Looi I., Dominguez J.C., Park K.W., Karanam A.K., Simon O. (2020). Role of fluid biomarkers and PET imaging in early diagnosis and its clinical implication in the management of Alzheimer’s disease. J. Alzheimer’s Dis. Rep..

[3] Cummings J., Lee G., Nahed P., Kambar M.E.Z.N., Zhong K., Fonseca J., Taghva K. (2022). Alzheimer’s disease drug development pipeline: 2022. Alzheimer’s Dement..

[4] Chen H., Xu J., Xu H., Luo T., Li Y., Jiang K., Shentu Y., Tong Z. (2023). New insights into Alzheimer’s disease: Novel pathogenesis, drug target and delivery. Pharmaceutics.

[5] Sweeney M.D., Sagare A.P., Zlokovic B.V. (2018). Blood-brain barrier breakdown in Alzheimer disease and other neurodegenerative disorders. Nat. Rev. Neurol..

[6] Rabinovici G.D., Gatsonis C., Apgar C., Chaudhary K., Gareen I., Hanna L., Hendrix J., Hillner B.E., Olson C., Lesman-Segev O.H. (2019). Association of Amyloid Positron Emission Tomography with Subsequent Change in Clinical Management Among Medicare Beneficiaries with Mild Cognitive Impairment or Dementia. JAMA.

[7] Thijssen E.H., La Joie R., Wolf A., Strom A., Wang P., Iaccarino L., Bourakova V., Cobigo Y., Heuer H., Spina A. (2020). Diagnostic value of plasma phosphorylated tau181 in Alzheimer’s disease and frontotemporal lobar degeneration. Nat. Med..

[8] Nakamura A., Kaneko N., Villemagne V.L., Kato T., Doecke J., Doré V., Fowler C., Li Q.X., Martins R., Rowe C. (2018). High performance plasma amyloid-β biomarkers for Alzheimer’s disease. Nature.

[9] Zhao Z., Nelson A.R., Betsholtz C., Zlokovic B.V. (2015). Establishment and dysfunction of the blood-brain barrier. Cell.

[10] van de Haar H.J., Burgmans S., Jansen J.F., van Osch M.J., van Buchem M.A., Muller M., Hofman P.A.M., Verhey F.R.J., Backes W.H. (2016). Blood-brain barrier leakage in patients with early Alzheimer disease. Radiology.

[11] Nation D.A., Sweeney M.D., Montagne A., Sagare A.P., D’Orazio L.M., Pachicano M., Sepehrband F., Nelson A.R., Buennagel D.P., Harrington M.G. (2019). Blood-brain barrier breakdown is an early biomarker of human cognitive dysfunction. Nat. Med..

[12] Tsukita S., Tanaka H., Tamura A. (2019). The claudins: From tight junctions to biological systems. Trends Biochem. Sci..

[13] Nitta T., Hata M., Gotoh S., Seo Y., Sasaki H., Hashimoto N., Furuse M., Tsukita S. (2003). Size-selective loosening of the blood-brain barrier in claudin-5-deficient mice. J. Cell Biol..

[14] Mandel I., Paperna T., Glass-Marmor L., Volkowich A., Badarny S., Schwartz I., Vardi P., Koren I., Miller A. (2012). Tight junction proteins expression and modulation in immune cells and multiple sclerosis. J. Cell. Mol. Med..

[15] Argaw A.T., Gurfein B.T., Zhang Y., Zameer A., John G.R. (2009). VEGF-mediated disruption of endothelial CLN-5 promotes blood-brain barrier breakdown. Proc. Natl. Acad. Sci. USA.

[16] Hashimoto Y., Shirakura K., Okada Y., Takeda H., Endo K., Tamura M., Watari A., Sadamura Y., Sawasaki T., Doi T. (2017). Claudin-5-binders enhance permeation of solutes across the blood-brain barrier in a mammalian model. J. Pharmacol. Exp. Ther..

[17] Berndt P., Winkler L., Cording J., Breitkreuz-Korff O., Rex A., Dithmer S., Rausch V., Blasig R., Richter M., Sporbert A. (2019). Tight junction proteins at the blood-brain barrier: Far more than claudin-5. Cell. Mol. Life Sci..

[18] Zlokovic B.V. (2011). Neurovascular pathways to neurodegeneration in Alzheimer’s disease and other disorders. Nat. Rev. Neurosci..

[19] Feng S., Cen J., Huang Y., Shen H., Yao L., Wang Y., Chen Z. (2011). Matrix metalloproteinase-2 and -9 secreted by leukemic cells increase the permeability of blood-brain barrier by disrupting tight junction proteins. PLoS ONE.

[20] Yang Y., Estrada E.Y., Thompson J.F., Liu W., Rosenberg G.A. (2007). Matrix metalloproteinase-mediated disruption of tight junction proteins in cerebral vessels is reversed by synthetic matrix metalloproteinase inhibitor in focal ischemia in rat. J. Cereb. Blood Flow Metab..

[21] Stewart P.A., Hayakawa K., Akers M.A., Vinters H.V. (1992). A morphometric study of the blood-brain barrier in Alzheimer’s disease. Lab. Investig..

[22] Kazmierski R., Michalak S., Wencel-Warot A., Nowinski W.L. (2012). Serum tight-junction proteins predict hemorrhagic transformation in ischemic stroke patients. Neurology.

[23] Kılıç F., Işık Ü., Demirdaş A., Doğuç D.K., Bozkurt M. (2020). Serum zonulin and claudin-5 levels in patients with bipolar disorder. J. Affect. Disord..

[24] Işık Ü., Aydoğan Avşar P., Aktepe E., Doğuç D.K., Kılıç F., Büyükbayram H. (2020). Serum zonulin and claudin-5 levels in children with obsessive-compulsive disorder. Nord. J. Psychiatry.

[25] Stewart P.A., Magliocco M., Hayakawa K., Farrell C.L., Del Maestro R.F., Girvin J., Kaufmann J.C., Vinters H.V., Gilbert J. (1987). A quantitative analysis of blood-brain barrier ultrastructure in the aging human. Microvasc. Res..

[26] Montagne A., Barnes S.R., Sweeney M.D., Halliday M.R., Sagare A.P., Zhao Z., Toga A.W., Jacobs R.E., Liu C.Y., Amezcua L. (2015). Blood-brain barrier breakdown in the aging human hippocampus. Neuron.

[27] Yamazaki Y., Shinohara M., Shinohara M., Yamazaki A., Murray M.E., Liesinger A.M., Heckman M.G., Lesser E.R., Parisi J.E., Petersen R.C. (2019). Selective loss of cortical endothelial tight junction proteins during Alzheimer’s disease progression. Brain.

[28] Andjelkovic A.V., Situ M., Citalan-Madrid A.F., Stamatovic S.M., Xiang J., Keep R.F. (2023). Blood-brain barrier dysfunction in normal aging and neurodegeneration: Mechanisms, impact, and treatments. Stroke.

[29] Morita K., Furuse M., Fujimoto K., Tsukita S. (1999). Claudin multigene family encoding four-transmembrane domain protein components of tight junction strands. Proc. Natl. Acad. Sci. USA.

[30] Scalise A.A., Kakogiannos N., Zanardi F., Iannelli F., Giannotta M. (2021). The blood-brain and gut-vascular barriers: From the perspective of claudins. Tissue Barriers.

[31] Greene C., Hanley N., Reschke C.R., Reddy A., Mäe M.A., Connolly R., Behan C., O’Keeffe E., Bolger I., Hudson N. (2022). Microvascular stabilization via blood-brain barrier regulation prevents seizure activity. Nat. Commun..

[32] Greene C., Kealy J., Humphries M.M., Gong Y., Hou J., Hudson N., Cassidy L.M., Martiniano R., Shashi V., Hooper S.R. (2018). Dose-dependent expression of claudin-5 is a modifying factor in schizophrenia. Mol. Psychiatry..

[33] Dion-Albert L., Cadoret A., Doney E., Kaufmann F.N., Dudek K.A., Daigle B., Parise L.F., Cathomas F., Samba N., Hudson N. (2022). Vascular and blood-brain barrier-related changes underlie stress responses and resilience in female mice and depression in human tissue. Nat. Commun..

[34] Viggiano D., Wagner C.A., Martino G., Nedergaard M., Zoccali C., Unwin R., Capasso G. (2020). Mechanisms of cognitive dysfunction in CKD. Nat. Rev. Nephrol..

[35] Qiu C., Winblad B., Marengoni A., Klarin I., Fastbom J., Fratiglioni L. (2006). Heart failure and risk of dementia and Alzheimer disease: A population-based cohort study. Arch. Intern. Med..

[36] Manemann S.M., Knopman D.S., St Sauver J., Bielinski S.J., Chamberlain A.M., Weston S.A., Jiang R., Roger V.L. (2022). Alzheimer’s disease and related dementias and heart failure: A community study. J. Am. Geriatr. Soc..

[37] Rosenkranz M.A., Dean D.C., Bendlin B.B., Jarjour N.N., Esnault S., Zetterberg H., Heslegrave A., Evans M.D., Davidson R.J., Busse W.W. (2022). Neuroimaging and biomarker evidence of neurodegeneration in asthma. J. Allergy Clin. Immunol..

[38] Rasmussen K.L., Tybjærg-Hansen A., Nordestgaard B.G., Frikke-schmidt R. (2019). Plasma levels of apolipoprotein E, APOE genotype, and all-cause and cause-specific mortality in 105,949 individuals from a white general population cohort. Eur. Heart J..

[39] Thorin E. (2015). Hypertension and Alzheimer disease: Another brick in the wall of awareness. Hypertension.

[40] Choi D., Choi S., Park S.M. (2018). Effect of smoking cessation on the risk of dementia: A longitudinal study. Ann. Clin. Transl. Neurol..

[41] Cirovic A., Cirovic A., Orisakwe O.E., Lima R.R. (2023). Local and systemic hypoxia as inductors of increased aluminum and iron brain accumulation promoting the onset of Alzheimer’s disease. Biol. Trace Elem. Res..

[42] Shen W., Li S., Chung S.H., Zhu L., Stayt J., Su T., Couraud P., Romero I.A., Weksler B., Gillies M.C. (2011). Tyrosine phosphorylation of VE-cadherin and claudin-5 is associated with TGF-β1-induced permeability of centrally derived vascular endothelium. Eur. J. Cell Biol..

[43] Awan F.M., Anjum S., Obaid A., Ali A., Paracha R.Z., Janjua H.A. (2014). In-silico analysis of claudin-5 reveals novel putative sites for post-translational modifications: Insights into potential molecular determinants of blood-brain barrier breach during HIV-1 infiltration. Infect. Genet. Evol..

[44] Annunziata P., Cioni C., Masi G., Tassi M., Marotta G., Severi S. (2018). Fingolimod reduces circulating tight-junction protein levels and in vitro peripheral blood mononuclear cells migration in multiple sclerosis patients. Sci. Rep..

[45] Wu H., Wang J., Teng T., Yin B., He Y., Jiang Y., Liu X., Yu Y., Li X., Zhou X. (2023). Biomarkers of intestinal permeability and blood-brain barrier permeability in adolescents with major depressive disorder. J. Affect. Disord..

[46] Usta A., Kılıç F., Demirdaş A., Işık Ü., Doğuç D.K., Bozkurt M. (2021). Serum zonulin and claudin-5 levels in patients with schizophrenia. Eur. Arch. Psychiatry Clin. Neurosci..

[47] Jongen P.J., Ter Horst A.T., Brands A.M. (2012). Cognitive impairment in multiple sclerosis. Minerva. Med..

[48] Yamamoto K.S., Utshigisawa T., Ogura H., Aoki T., Kawakami T., Ohga S., Ohara A., Ito E., Yamamoto T., Kanno H. (2022). Clinical and genetic diagnosis of thirteen Japanese patients with hereditary spherocytosis. Hum. Genome Var..

[49] Tachibana K., Hashimoto Y., Shirakura K., Okada Y., Hirayama R., Iwashita Y., Nishino I., Ago Y., Takeda H., Kuniyasu H. (2021). Safety and efficacy of an anti-claudin-5 monoclonal antibody to increase blood-brain barrier permeability for drug delivery to the brain in a non-human primate. J. Control. Release.

[50] Fukasawa M., Nagase S., Shirasago Y., Iida M., Yamashita M., Endo K., Yagi K., Suzuki T., Wakita T., Hanada K. (2015). Monoclonal antibodies against extracellular domains of claudin-1 block hepatitis C virus infection in a mouse model. J. Virol..

[51] Hashimoto Y., Zhou W., Hamauchi K., Shirakura K., Doi T., Yagi K., Sawasaki T., Okada Y., Kondoh M., Takeda H. (2018). Engineered membrane protein antigens successfully induce antibodies against extracellular regions of claudin-5. Sci. Rep..

[52] Zhou W., Takeda H. (2020). Cell-free production of proteoliposomes for functional analysis and antibody development targeting membrane proteins. J. Vis. Exp..

[53] Wilson D.H., Rissin D.M., Kan C.W., Fournier D.R., Piech T., Campbell T.G., Meyer R.E., Fishburn M.W., Cabrera C., Patel P.P. (2016). The Simoa HD-1 Analyzer: A novel fully automated digital immunoassay analyzer with single-molecule sensitivity and multiplexing. J. Lab. Autom..

