# Supplementary material for: Association of Plasma Claudin-5 with Age and Alzheimer Disease
Source: Int J Mol Sci. 2024 Jan 24;25(3):1419. doi: 10.3390/ijms25031419 (PMC10855409; doi:10.3390/ijms25031419)
Supplement: Supplementary file 1 [file ijms-25-01419-s001.zip › ijms-2829160-supplementary.pdf]

International Journal of Molecular Sciences

Supplementary Material

Association of plasma claudin-5 with age and Alzheimer disease

Keisuke Tachibana [1], Ryuichi Hirayama [2], Naoyuki Sato [2,3], Kotaro Hattori [4], Takashi Kato [5], Hiroyuki Takeda [6] and Masuo Kondoh [1,*]

[1] Graduate School of Pharmaceutical Sciences, Osaka University, Osaka, Japan

[2] Graduate School of Medicine, Osaka University, Osaka 565-0871, Japan

[3] Department of Aging Neurobiology, Center for Development of Advanced Medicine for Dementia, Na-tional Center for Geriatrics and Gerontology, Aichi 474-8511, Japan

[4] Medical Genome Center, National Institute of Neuroscience, National Center of Neurology and Psychi-atry, Kodaira, Tokyo 187-8502, Japan

[5] Department of Clinical and Experimental Neuroimaging, National Center for Geriatrics and Gerontology, Aichi 474-8511, Japan

[6] Proteo-Science Center, Ehime University, Ehime 790-8577, Japan

[*] Correspondence: masuo@phs.osaka-u.ac.jp; Tel/Fax: 81-6-6879-8195.

A.

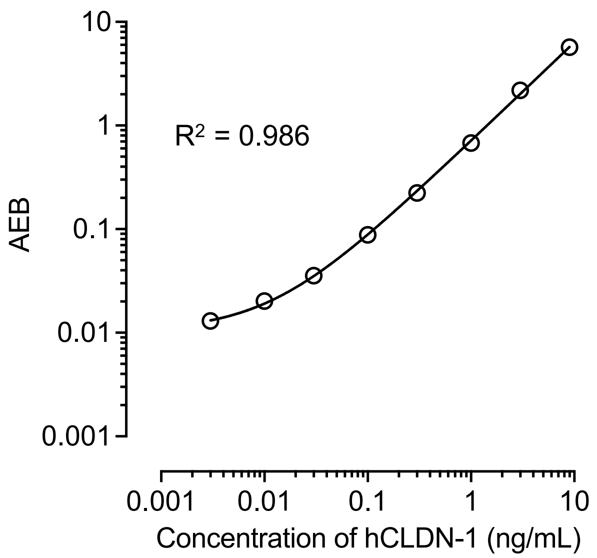

B.

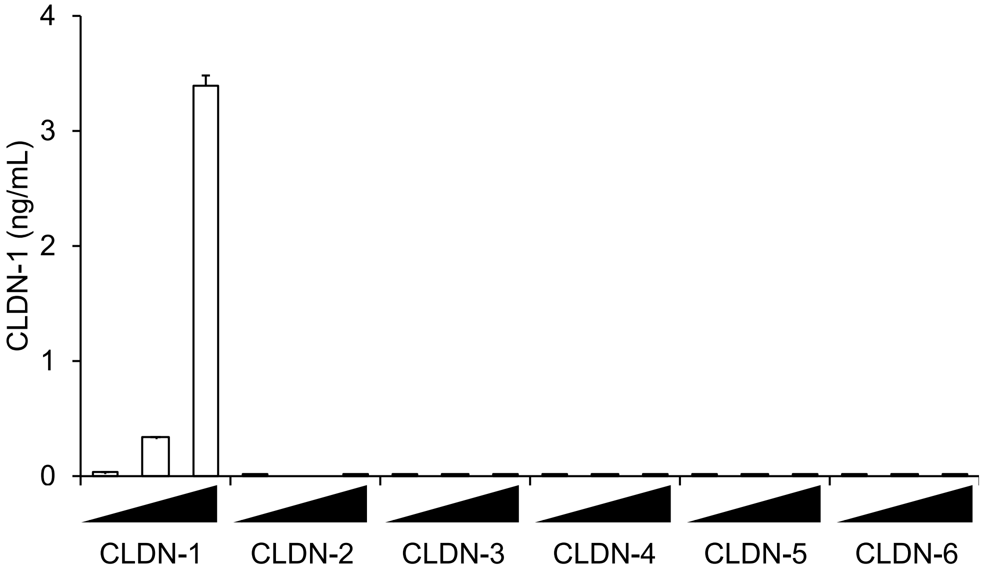

Supplementary Figure S1. Sensitivity and specificity of a plasma human claudin (CLDN) 1 protein detection system using Simoa. (A) Response curve of the Simoa assay for the detection of human CLDN-1. Data are given as mean ± standard deviation (n = 5). Standard curves were generated by using a four-parameter logistic model, and R-squared values were calculated. (B) Analysis of the specificity of CLDN-1 detection using Simoa. Proteoliposomes containing human CLDN-1 to -6 were tested at concentrations of 0.03, 0.3, and 3 ng/mL. Data are given as mean ± standard deviation (n = 3).

Supplementary Table S1. Analysis of the sensitivity and specificity of the Simoa-based human claudin (CLDN) 5 protein detection system. This table contains the data used for the graphs in Figure 1C (n = 3).

**CLDN**	**Theoretical concentration (ng/mL)**	**Actual concentration (ng/mL)**	
		**Mean**	**SD**
CLDN-1	0.03	0.002	0.001
	0.3	0.001	0.000
	3	0.001	0.000
CLDN-2	0.03	0.002	0.000
	0.3	0.002	0.000
	3	0.001	0.001
CLDN-3	0.03	0.001	0.000
	0.3	0.001	0.000
	3	0.001	0.000
CLDN-4	0.03	0.002	0.000
	0.3	0.002	0.000
	3	0.002	0.000
CLDN-5	0.03	0.035	0.004
	0.3	0.365	0.005
	3	3.622	0.014
CLDN-6	0.03	0.001	0.000
	0.3	0.001	0.001
	3	0.001	0.000

SD, standard deviation.

Supplementary Table S2. Precision and accuracy of quantification of human CLDN-1 concentration series.

Theoretical concentration (ng/mL)	Actual concentration (ng/mL)					Mean (ng/mL)	SD	CV (%)	Accuracy
	1	2	3	4	5				
0	0.002	ND	0.000	ND	0.001	0.001	0.001	95.79	NA
0.003	0.003	0.003	0.004	0.004	0.000	0.003	0.002	53.96	94.84
0.01	0.010	0.015	0.009	0.012	0.011	0.011	0.002	18.59	114.03
0.03	0.030	0.031	0.029	0.030	0.032	0.030	0.001	3.84	101.07
0.1	0.097	0.101	0.108	0.091	0.099	0.099	0.006	6.09	99.31
0.3	0.281	0.271	0.292	0.293	0.289	0.285	0.009	3.17	95.12
1	0.998	0.914	0.914	0.938	0.952	0.943	0.035	3.69	94.31
3	3.311	3.129	3.137	3.411	3.275	3.253	0.120	3.69	108.42
9	9.549	9.216	9.488	7.058	9.274	8.917	1.049	11.76	99.08

CV, coefficient of variation; NA, not applicable; ND, not detected; SD, standard deviation.

Supplementary Table S3. Analysis of the sensitivity and specificity of the Simoa-based human claudin (CLDN) 1 protein detection system. This table contains the data used for the graphs in Supplementary figure S1B (n = 3).

CLDN	Theoretical concentration (ng/mL)	Actual concentration (ng/mL)	
		Mean	SD
CLDN-1	0.03	0.031	0.002
	0.3	0.330	0.006
	3	3.386	0.092
CLDN-2	0.03	0.001	NA
	0.3	ND	NA
	3	0.001	0.000
CLDN-3	0.03	0.000	NA
	0.3	0.000	0.000
	3	0.000	NA
CLDN-4	0.03	0.000	0.000
	0.3	0.002	NA
	3	0.002	NA
CLDN-5	0.03	0.001	0.001
	0.3	0.001	0.001
	3	0.001	0.001
CLDN-6	0.03	0.001	NA
	0.3	0.001	0.000
	3	0.001	0.001

NA, not applicable; ND, not detected; SD, standard deviation.
